# Human papillomavirus genotyping using next generation sequencing (NGS) in cervical lesions: Genotypes by histologic grade and their relative proportion in multiple infections

**DOI:** 10.1371/journal.pone.0278117

**Published:** 2022-11-23

**Authors:** Jorge Alejandro Basiletti, Joan Valls, Tomás Poklépovich, María Dolores Fellner, Maryluz Rol, Rafael Alonso, Rita Mariel Correa, María Celeste Colucci, Mercedes Rodríguez de la Peña, Paula Gabriela Falabella, Agustina Saíno, Josefina Campos, Rolando Herrero, Maribel Almonte, María Alejandra Picconi

**Affiliations:** 1 Servicio Virus Oncogénicos, Laboratorio Nacional y Regional de Referencia de HPV, Instituto Nacional de Enfermedades Infecciosas-ANLIS “Dr. Malbrán”, Buenos Aires, Argentina; 2 Early Detection, Prevention & Infection Branch, International Agency for Research on Cancer, World Health Organization, Lyon, France; 3 Centro de Investigación Biomédica en Red de Cáncer (CIBERONC), Madrid, Spain; 4 Unidad Operativa Centro de Genómica y Bioinformática, ANLIS "Dr. Malbrán", Buenos Aires, Argentina; 5 Departamento de Métodos Cuantitativos, Facultad de Medicina, Universidad de la República, Montevideo, Uruguay; 6 Servicio de Ginecología, Hospital Nacional “Prof. Posadas”, El Palomar, Provincia de Buenos Aires, Argentina; 7 Servicio de Anatomía Patológica, Hospital Nacional “Prof. Posadas”, El Palomar, Provincia de Buenos Aires, Argentina; 8 Agencia Costarricense de Investigaciones Biomédicas, San José, Costa Rica; University of Ferrara: Universita degli Studi di Ferrara, ITALY

## Abstract

Sensitive and specific genotyping of human papillomaviruses (HPVs) is critical for the surveillance and monitoring of the vaccine effectiveness. Here, HPV genotypes were identified in 137 cervical samples with different histology (79 ≤CIN1 and 58 CIN3+) using Nested-PCR followed by Next-Generation sequencing (NGS) and relative proportions for each genotype in multiple infections were computed. All samples had been previously genotyped by PCR-Reverse Blotting Hybridization (PCR-RBH) thus allowing for a concordance analysis between both techniques. Multiple infections were present in 85% of ≤CIN1 cases compared to only 41% in CIN3+ cases (p<0.001). Among ≤CIN1 cases a towering genotypic diversity was observed, considering both low (LR-) and high risk (HR-) HPV genotypes; while among CIN3+, diversity was lower, HR-HPVs prevailing in most cases, especially HPV16. Furthermore, the predominance of HR-HPV genotypes in the proportions identified in each sample was higher in CIN3+ cases [(HPV16 (62.5%), followed by HPV31 and HPV58 (8.3% each)], than in ≤CIN1 cases [(HPV16 (17.7%), followed by HPV52 (14.7%) and HPV31 (10.3%)]. Agreement between PCR-RBH and NGS was higher than 90% for all genotypes (with an overall Kappa of 0.7), even though NGS identified eighty-nine positive results for HPV genotypes that had not been detected by PCR-RBH, evidencing its greater sensitivity. These results suggest that a reduction in genotypic diversity and/or an increase in the relative proportion of HR-HPVs in multiple infections can be considered as a biomarker for the potential risk of malignant progression.

## Introduction

More than 200 genotypes of Human Papillomavirus (HPV) have been completely characterized, and new HPV types are still being found [1, 2]. Among them, 40 genotypes are known to infect the anogenital and aerodigestive tracts, and are grouped into high risk (HR)-HPV and low risk (LR)-HPV types based on their oncogenic potential [3, 4].

Classification of HPVs is based on the nucleotide sequence homology of the L1 gene, which is the most conserved region of the viral genome. Within the family, different genera share less than 60% nucleotide similarity. Within each genus, different species share similarity between 60% and 70%. Below the species level, a novel HPV type shares less than 90% similarity to any other type [1, 5]. The definition of a variant lineage is that the L1 open-reading frame differs by more than 1%, but less than the 10% that would make it another HPV type [6]. The International Agency for Research on Cancer (IARC) classifies twelve HPV genotypes as *carcinogenic* (HR-HPV) (Group 1: HPVs 16, 18, 31, 33, 35, 39, 45, 51, 52, 56, 58, 59), whereas HPV68 is *probably carcinogenic* (probable HR-HPV) (Group 2A) and HPVs 26, 53, 66, 73 and 82 are *possibly carcinogenic* (possible HR-HPV) (Group 2B); the so-called low-risk (LR) types (HPV6, 11, 40, 42, 43, 44, 55, 61, 81, 83) show strong evidence that they do not cause cancer (Group 4) [4, 7, 8].

Persistent HR-HPV infection is the main cause of cervical cancer (CC) [9], with HPV16 and HPV18 persistence accounting for over 70% of cases [10–15]. HPV has also been detected in other anatomic sites. causing oropharyngeal, vulvar, vaginal, penile and anal cancers [9, 16–18].

Viral genotyping for epidemiological and surveillance studies and vaccine trials needs highly sensitive, high-throughput and affordable HPV detection assays. Consensus or degenerate primers are widely used for broad-spectrum HPV PCR amplification. Their high sensitivity and robust format have allowed their wide use globally. However, they have some limitations, mainly due to the different performance of each genotype amplification, especially in the case of multiple infections, since the viruses present in a lower proportion could result in false negatives. The methods used for genotyping the amplicons often count on hybridization of individual type-specific probes (corresponding to the most relevant genotypes already characterized), allowing multiple types to be detected simultaneously despite a wide variation in the viral load and amplification bias [19–24].

DNA sequencing by Sanger has the potential to identify a wide range of HPV types in simple infections. However, in multiple infections, its reading accuracy decreases due to the overlapping of electropherograms, and requires prior cloning, which is laborious and time-consuming; in addition, minority genotypes that are part of mixed infections might not be detected.

More recently, the emergence of Next Generation Sequencing (NGS) has brought an innovative and attractive solution since it allows to detect a larger spectrum of HPV genotypes. This high-throughput methodology relies on massive and parallel sequencing of single molecules from small amounts of DNA. Therefore, it provides an enormous amount of information, allowing to identify all the genotypes present in a sample, regardless of the viral load and the characteristics of its sequence.

Although the prevalence and distribution of HPV genotypes in the cervix have been extensively studied, deep genomic analyses in cervical lesions with a different histological severity, regarding the diversity, abundance, and characteristics of the HPV genotypes are still scarce. NGS reports have shown that previous studies applying more limited techniques underestimated certain viral types and missed new genotypes, which could have an impact on pathogenesis, molecular epidemiology, virological surveillance and eventually on screening using HPV tests, particularly in vaccinated populations [25–27].

Moreover, the benefit is even greater when analysing multiple infections, whose implications in cervical carcinogenesis are still controversial [28–31]. In samples with coinfections, where genotype identification is complex and occasionally imprecise, NGS provides a powerful lens through which to look at viral communities in detail that would be unnoticeable with conventional methods, allowing to evaluate the proportion of each genotype in the viral mixture. Some authors have determined a greater average abundance of HR-HPVs in HSIL [25], but data are still limited in histology-confirmed cervical lesions.

This study aimed to identify HPV genotypes with NGS in cervical samples with different histologies, and to determine the relative proportion of each genotype within each sample in multiple infections.

## Materials and methods

### Study design

**Fig 1:**
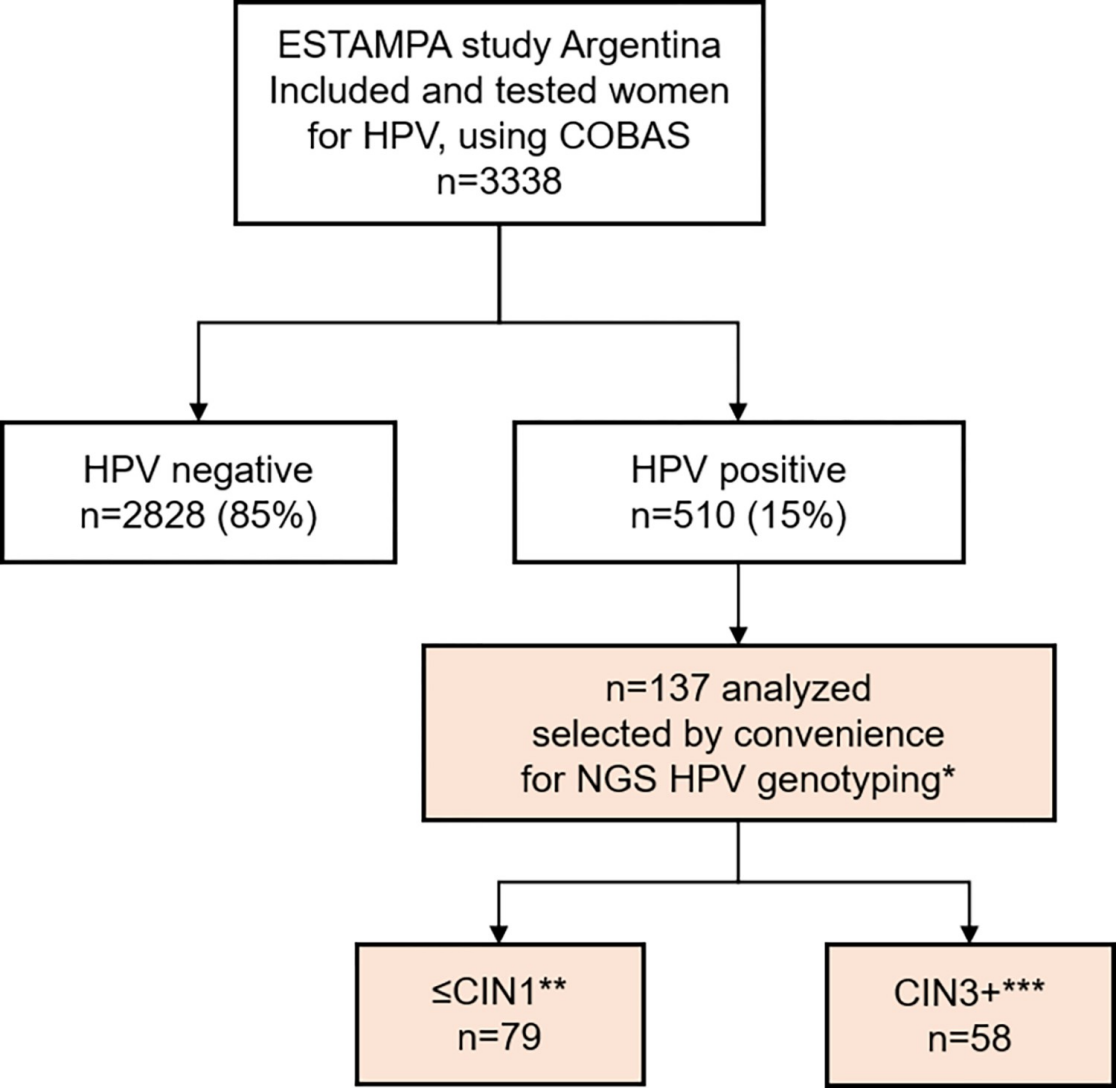
Selection of the study population. *All 137 samples had previously been genotyped for PCR/RBH. **≤CIN1: Normal Cytology (Negative for Intraepithelial lesion or Malignancy, NILM) and CIN1. ***CIN3+: CIN3 and CC.

This is a cross-sectional, observational study, ancillary of the multicentric ESTAMPA study (Spanish acronym for “EStudio multicéntrico de TAMizaje y triaje del cáncer cervicouterino con pruebas de virus PApiloma humano”) [32] to assess the HPV-genotype distribution using NGS including a series of 137 women selected from Argentina (Fig 1).

### Clinical samples

Our series for the present study included 137 cervical samples collected at the initial visit of ESTAMPA Argentina, classified into two groups according to histology results: (1) ≤CIN1 (n = 79), which included 39 normal cytology (NILM) or negative histology and 40 CIN1, selected by convenience sampling and (2) CIN3+ (n = 58), which included 54 CIN3 and 4 CC (all the CIN3+ diagnosed were considered in this group).

All samples had been previously genotyped separately by two PCR strategies combined with Reverse Blotting Hybridization (RBH) using type-specific biotinylated probe sets [33]: i) Broad-Spectrum General Primers 5+/6+ PCR (BSGP) [24, 34] and ii) PGMY9/11 (CHUV) [23, 35], which allow to amplify highly conserved 140 bp and 450 bp fragments of the HPV-L1 gene, respectively [45]. These “in house” techniques coupled PCR amplification to hybridization to specific probes for 37 anogenital HPV genotypes (LR and HR) immobilized on a nylon membrane. These assays have been analytically validated by their developers [23, 24, 34, 35] and verified by us upon implementation in our laboratory; they are usually used in many laboratories for HPV genotyping due to their highly valuable sensitive for HPV epidemiological studies and surveillance [33, 36–40]. The Regional HPV Reference Laboratory from Argentina annually participates in the external control of HPV genotyping (HPV DNA typing proficiency panel), provided by the International Papillomavirus Reference Centre, which adds reliability to the results obtained [41].

### HPV genotyping by NGS

#### Analytical characterization

To determine the sensitivity and specificity of the NGS genotyping protocol applied in this study, 20 ng of genomic DNA + 5, 50 and 500 copies per 5ul of HPV16, HPV18, and 50 and 500 copies per 5ul of HPV31, HPV33, HPV35, HPV39, HPV45, HPV51, HPV58, HPV59, HPV68, HPV6 and HPV11 plasmid and HPV negative cell lines (C33A cells) were used as reference samples. The different HPV types were combined in various amounts to mimic multiple infections.

#### PCR amplification

Was performed by a nested PGMY/GP primer set, using 5ul of each plasmid sample and 20 ng of DNA previously extracted from cervical cells collected in ThinPrep PreservCyt Solution (Hologic, Bedford, MA, USA) with commercial columns on a robotic system (QIAcube system, Qiagen). Briefly, two successive rounds of PCR were carried out to obtain a final target sequence of about 200 bp length of the L1 gen. The first round of PCR with a mixture of primers for PGMY11/09 [20] and the second round with nested HPV PCR primers GP5+/GP6+ [24, 34, 42].

#### HPV DNA sample library preparation

Amplicons were purified using DNA Clean & Concentrator (Zymo Research Corp.), according to manufacturer´s instructions. Purified DNA concentration was determined using a Qubit fluorometer (Life Technologies Corporation, Carlsbad, CA, USA). DNA libraries were prepared using an Illumina-compatible kit (NEBNext® Ultra™ II DNA Library Prep Kit, NEB, Frankfurt am Main, Germany), following the manufacturer´s recommendations (New England BioLabs, Herts, UK). Briefly, purified amplicons were end-repaired, adaptor-ligated, and cleaned up. Subsequently, DNA libraries obtained from each sample were amplified by nested-PCR with different index primers and then purified using Am-Pure XP beads (Beckman Coulter, Porterville, CA, USA). DNA libraries with different index primers were pooled in equal amounts.

#### Sequencing using the MiSeq platform

Paired-end (150×2) deep sequencing was performed using the MiSeq v2 reagent kit (Illumina, Inc., San Diego, CA, USA) on the MiSeq platform (Illumina, Inc.) with a standard protocol.

#### Bioinformatics

The FASTQ data went through a first processing to exclude the low quality reads (Q-score <30) and the adapter sequences, by cutting regions with the FastQC [43] and Trimgalore software [44]. The presence of HPV and potential contaminants reads, such as human DNA, were identified with Kraken 2 software [45]. Trimmed sequences were mapped into human genome hg38 using Bowtie [46]. Reads with at least 95% identity and 75% coverage of human DNA were removed. Only unmapped paired-end reads were assembled to generate about 180 bp L1 contigs using Trinity software [47] and then, consensus sequences were generated from contigs with high similarity (pairwise distance ≤3%). Consensus L1 sequences were aligned to L1 region of HPV reference sequences obtained from the NCBI and Papillomavirus Episteme PaVE databases (http://pave.niaid.nih.gov) using Bowtie [46]. Only the consensus sequences that, when compared to the PaVE base, showed at least 90% identity and >70% coverage, were considered positive for one HPV genotype.

HPV genotypes were identified for each sample (as present or absent) and their relative presence further quantified based on the number of reads matched to each consensus genotype in relation to the total number of reads, allowing to evaluate the relative proportions of each genotype within a sample. The weeSAM software [48] was used to count the mapped reads on each fragment.

Therefore, absolute frequencies for each genotype were used first to compute their prevalence within histological grade. Second, the proportion of each genotype present in the samples with multiple infections was determined as the ratio of the number of reads corresponding to each genotype by the total number of reads in each sample. Third, the prevalence of predominant genotypes, i.e. that with the highest observed proportion, among samples where the genotype was present, was computed within histologic grade. In addition, the proportions were also used to compute a Gini index as a measure of genotypic diversity for each sample; a heatmap was produced to visualize these proportions within samples by histologic grade and sorting them out with the Gini index.

NGS genotyping results were compared with those previously obtained by PCR-RBH in the same samples. This analysis was restricted to the 37 HPV genotypes detectable by all RBH probes.

### Ethical considerations

The ESTAMPA protocol was approved by the Ethics Committee of the International Agency for Research on Cancer (IEC Project 12–27-A7), the Pan American Health Organization (PAHO) Ethical Committee and Ethical Committees of the Hospital Nacional “Prof. Posadas” (Argentina). The participants signed an informed consent that included details on the background, study procedures, risks and benefits, statement of confidentiality, specimen use and study staff to contact.

### Statistical analyses

A chi-square test was used to compare the distribution of single and multiple infections by lesion severity. A Student t-test was used to assess whether genotypic diversity, as measured with the Gini index, was associated to histologic grade. Cohen’s Kappa was used to globally assess the concordance between NGS and PCR-RBH-genotyping in the positivity for any genotype. To further investigate the concordance at the genotype level, an overall agreement was calculated for each HPV type as the sum of the number of specimens positive by both assays plus the number of negative ones by both assays, divided by the total number of specimens tested multiplied by 100, using McNemar test to evaluate the differences. In addition, positive agreement was also presented, i.e. excluding those that were negative for both tests. Threshold for statistical significance was set at p<0.05.

## Results

One hundred thirty-seven cervical samples were sequenced by NGS and bioinformatically analysed for HPV detection.

When using a cut-off value of 50 reads, HPV types in plasmid samples and HPV negative cell lines were correctly detected and typed with NGS at different DNA amounts (5, 50 and 500 copies/5μL for HPV16 and HPV18; 50 and 500 copies/5μL for the other HPV types). Therefore, a threshold of 50 reads was set for positive samples. Furthermore, all samples showed complete agreement for all types, even when containing multiple infections.

The HPV aligned reads in HPV positive samples showed a median of 1267 (minimum: 50; maximum: 31983) in the ≤CIN1 group, while in the CIN3+ group the median was 5507 (minimum: 51; maximum: 26783) (S1 Table).

**Fig 2:**
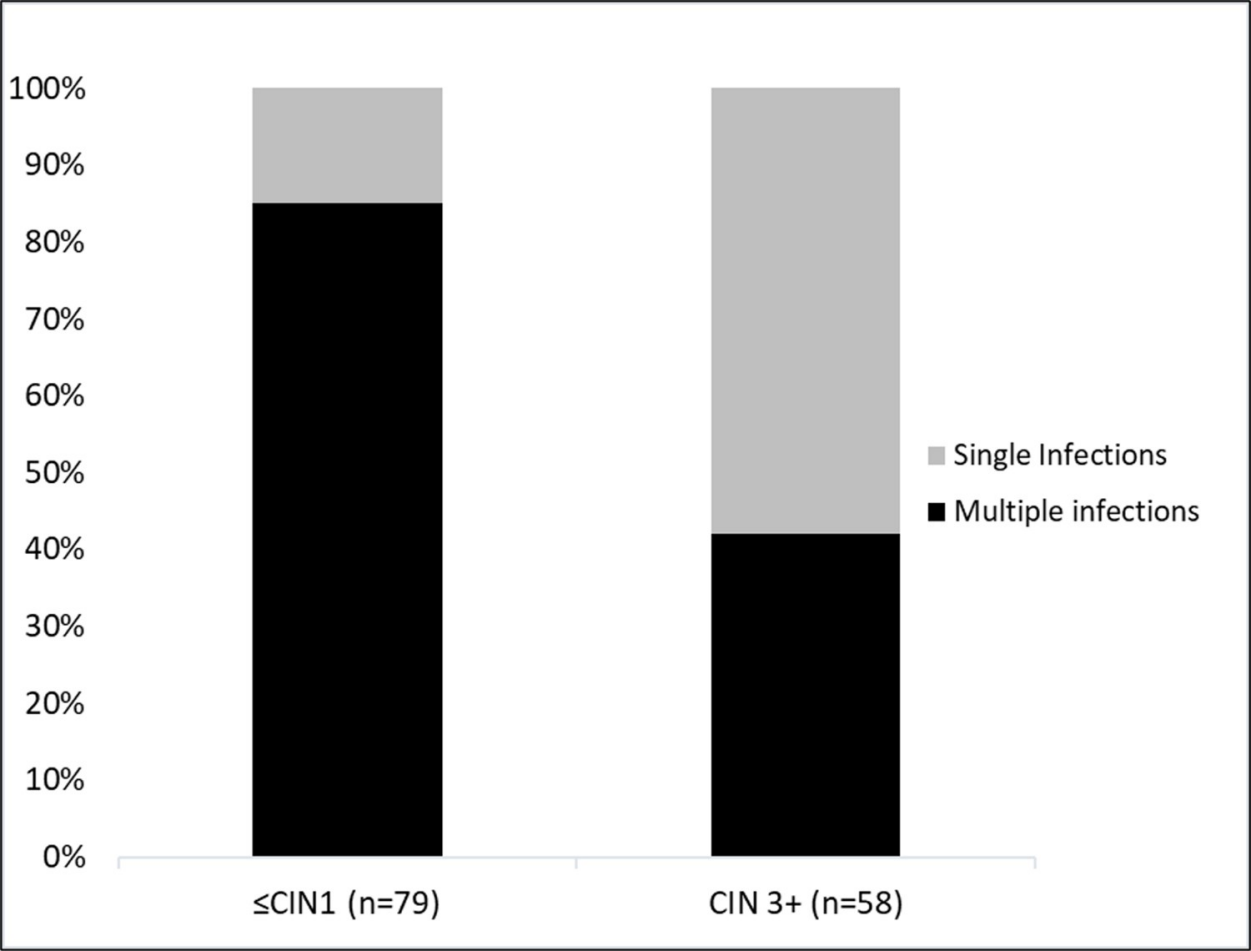
Distribution of single and multiple infections, according to histology.

Fig 2 illustrates the distribution of single and multiple infections by lesion severity. In the ≤CIN1 group, multiple infections stand for 85% of cases, whereas in the CIN3+ cases they encompass 41% (p<0.001); therefore, fewer multiple infections were detected in the higher severity cervical lesions.

**Fig 3:**
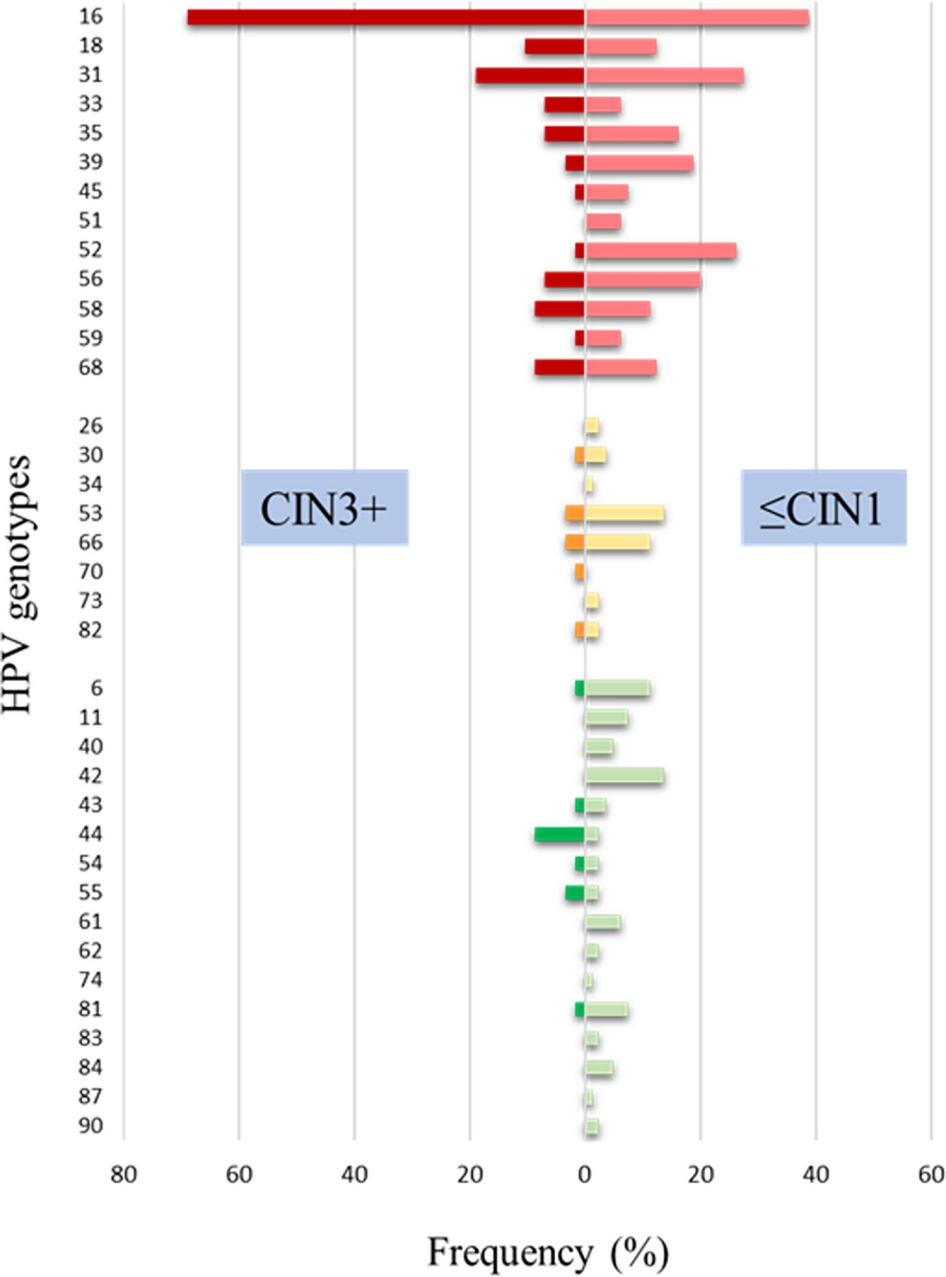
Distribution of HPV genotypes by severity of the lesion. HR-HPVs (Group 1 IARC) are in red, possible or probable HR-HPVs (Group 2A/2B IARC) are in orange and low-risk HPVs (Group 4 IARC) are in green.

Fig 3 details the frequencies of the 37 genotypes identified by NGS for each histological category. In case of multiple infections, each genotype was counted independently. HPV16 was the most prevalent genotype in both groups. The ten more frequent genotypes, in descending order were: for ≤CIN1: HPV16 (38.8%), HPV31 (27.5%), HPV52 (26.3%), HPV56 (20%), HPV39 (18.8%), HPV35 (16.3%), HPV18 (12.5%), HPV68 (12.5%), HPV58 (11.3%), HPV45 (7.5%), and for CIN3+: HPV16 (69%), HPV31 (19%), HPV53 (13.8%), HPV66 (11.3%), HPV18 (10.3%), HPV58 (8.6%), HPV68 (8.6%), HPV33 (6.9%), HPV35 (6.9%), and HPV56 (6.9%).

Our results showed a higher genotype diversity in the ≤CIN1 group, both HR and LR-HPV, while in CIN3+; the viral spectrum was more restricted, detecting almost exclusively HR-HPVs, particularly HPV16.

**Fig 4:**
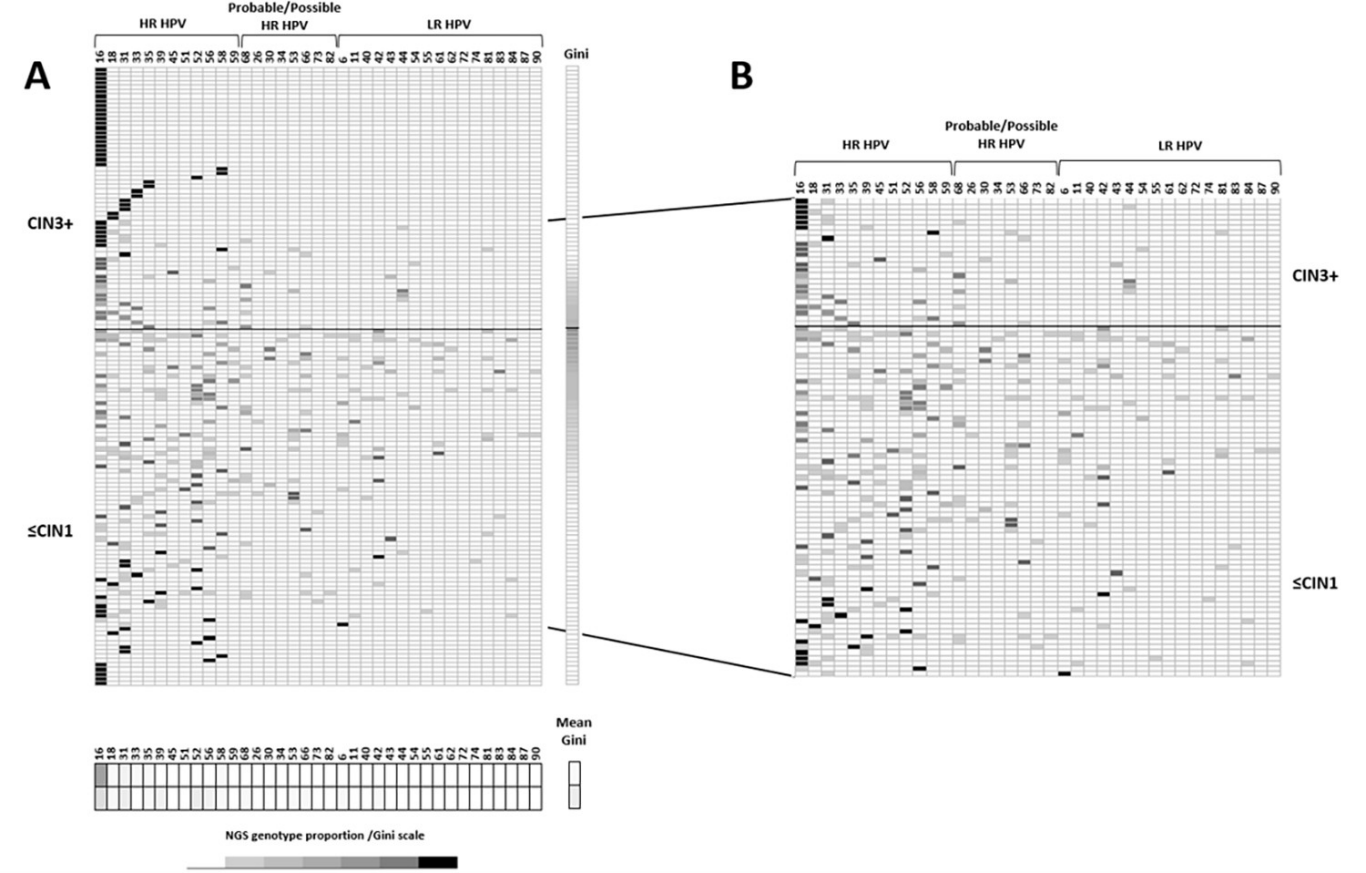
HPV genotypic diversity and relative proportion of HPV genotypes in cervical samples by NGS, according histology. HPV genotypes were classified according IARC criteria from carcinogenic (Group 1), probably/possibly carcinogenic (Group 2A/B) to probably not carcinogenic (Group 4). Lines correspond to each of the studied samples, the columns to each genotype identified and the different shades of gray in the cells represent the relative proportion, in percentage, of each genotype detected within a sample (the lightest corresponding to the lowest proportion and the darkest to the highest proportion). A: Individual and mean Gini coefficient to measure genotypic diversity are shown for each sample and histological group, respectively (79 ≤CIN1 and 58 CIN3+). Samples grade are ordered by Gini coefficient, from minimal (white) to maximal (black) genotypic diversity. B: Zoom in showing the HPV genotypic relative proportions in 92 cervical samples (68 ≤CIN1 and 24 CIN3+) with multiple infections.

The analysis of the relative proportion of the genotypes identified in each sample is detailed in the heatmap (Fig 4A). According to the severity of the lesion different patterns were observed: in ≤CIN1, a wide diversity of HPV genotypes, both LR and HR, was observed in different relative proportions in each sample, without a marked predominance of any of them; while the CIN3+ group exhibits a sharp drop in the diversity of genotypes with a notable predominance of HR-HPVs, especially HPV 16. Notably, mean Gini index was significantly higher in <CIN1 samples, showing a 2.2-fold increase in genotypic diversity when compared to CIN3+ samples (0.20±0.22 vs 0.09±0.17 respectively, p = 0.001). Genotypic diversity Fig 4B shows in greater detail the situation in the 92 multiple infections samples. In the ≤CIN1 group, the relative proportions of genotypes found in each sample do not show a marked predominance of one or some of them, the highest relative proportion corresponding to HPV16 (17.7%), followed by HPV52 (14.7%) and HPV31 (10.3%); while in the CIN3+ group, a worthy superiority in the relative proportions of HR-HPV stands out, the predominance being HPV16 (62.5%), followed by HPV31 and HPV58 (8.3% each).

Results of the HPV genotypes identified by RBH and NGS in the 137 cervical samples are shown in Table 1, reporting a good concordance both when genotypes were considered individually and as a whole group (Kappa 0.70). The overall agreement was higher than 90% for all genotypes although the positive agreement showed higher variations suggesting less concordance between the two assays when positivity was present. However, NGS identified eighty-nine new positive results for genotypes included in the RBH probe pool which had not been detected by PCR-RBH. Among them, forty-eight corresponded to ten HR-HPVs (HPVs 16, 18, 31, 33, 35, 39, 45, 51, 52 and 56), eight were probable HR-HPV (HPV 68), nine were possible HR-HPVs (HPVs 26, 53 and 66), and twenty-four corresponded to nine LR-HPVs (HPVs 6, 11, 40, 42, 43, 55, 61, 70 and 81). Differences in agreement were statistically significant for HPVs 16, 31, 35, 39 and 68 (McNemar’s p <0.05).

**Table 1:** Comparison of identified HPV genotypes by RBH and NGS on the 137 cervical samples. The analysis was restricted to the 37 genotypes which can be detected by both methods.

HPV Genotypes	No. of specimens						% Agreement	% Positive Agreement	McNemar P Value
	RBH positive	NGS positive	RBH-NGS-	RBH+ NGS-	RBH-NGS+	RBH+ NGS+			
**6**	5	10	127	0	5	5	96	50	0,07
**11**	2	6	131	0	4	2	97	33	0,13
**16**	64	71	66	0	7	64	95	90	**0.02**
**18**	13	16	121	0	3	13	98	81	0,25
**26**	1	2	135	0	1	1	99	50	1,00
**31**	21	33	104	0	12	21	91	64	**0,001**
**33**	6	9	128	0	3	6	98	67	0,25
**34**	1	1	135	1	1	0	99	0	0,48
**35**	11	17	120	0	6	11	96	65	**0,04**
**39**	9	17	120	0	8	9	94	53	**0,01**
**40**	3	4	133	0	1	3	99	75	1,00
**42**	9	11	126	0	2	9	99	82	0,48
**43**	2	4	133	0	2	2	99	50	0,48
**44**	7	7	126	4	4	3	94	27	0,72
**45**	6	7	130	0	1	6	99	86	1,00
**51**	4	5	132	0	1	4	99	80	1,00
**52**	18	22	115	0	4	18	97	82	0,88
**53**	9	13	123	1	5	8	96	57	0,22
**54**	8	3	128	6	1	2	95	22	0,13
**55**	3	4	132	1	2	2	98	40	1,00
**56**	17	20	114	3	6	14	93	61	0,50
**58**	16	14	121	2	0	14	99	88	0,48
**59**	9	6	127	4	1	5	96	50	0,37
**61**	1	5	132	0	4	1	97	20	0,13
**66**	7	11	126	0	4	7	97	64	0,13
**57**	0	0	137	0	0	0	100	NA	N/A
**68**	7	15	122	0	8	7	94	47	**0,01**
**69**	0	0	137	0	0	0	100	NA	N/A
**70**	0	1	136	0	1	0	99	0	1,00
**71**	0	0	137	0	0	0	100	NA	N/A
**72**	0	0	137	0	0	0	100	NA	N/A
**73**	4	2	132	3	1	1	97	20	0,62
**81**	3	7	130	0	4	3	97	43	0,13
**82**	4	3	132	2	1	2	98	40	1,00
**83**	2	2	135	0	0	2	100	100	N/A
**84**	4	4	129	4	4	0	94	0	0,72
**89**	1	0	136	1	0	0	99	0	1,00

On the other hand, RBH yielded eight positive results for HPVs 58 and 59 (HR-HPVs) and HPVs 73 and 82 (probable HR-HPV), which were not obtained by NGS, but they lacked statistical significance.

Additionally, NGS allowed to identify five genotypes (HPVs 30, 62, 74, 87 and 90), beyond the 37 probes included in the RBH assay.

## Discussion

For optimal epidemiological and surveillance studies and vaccine trials, it is essential to as far as possible identify all genotypes present in the sample, individually, and avoid false-negative results. This work takes advantage of the deep analysis of the nucleic acid sequences using NGS to expand the information about the HPV genotypes’ distribution among the histologically characterized cervical samples, and to explore the proportion of each genotype in coinfections.

In agreement with previous publications, HPV16 was by far the most frequently detected type in all histological grades [11, 49, 50]; moreover, our data also add evidence on the strong increase of its relative contribution compared to other HR-HPVs with disease severity from ≤CIN1 (38.8%) to CIN3+ (69.0%).Our series, albeit small, reproduces the broad spread of HPV16 at global level that was already well established in numerous and large studies [10, 14, 50, 51], indicating a substantial advantage over all other mucosal HPV types in terms of transmissibility and/or persistence.

In our series, the ≤CIN1 group, exhibited the greatest genotype diversity, both HR and LR-HPV (36 genotypes identified). A recent study from our group [33] assessed the genotypic diversity with PCR-RHB genotyping, using an extension of the series analysed here and also showing higher genotypic diversity in ≤CIN1 than in CIN3 both for HR and LR HPVs genotypes. Our findings, in the Latin American region, are consistent with what has been already described in the literature [14, 52] in other regions of the world. It confirms the broad spread of these viruses in the sexually active population. On the other hand, in CIN3+, the viral spectrum was much more restricted (23 genotypes identified), detecting almost exclusively HR-HPVs, as previously reported [10–15]. The inverse correlation between HPV diversity and progressive disease is also consistent with the findings of 1,518 cervical biopsies ranging from negative to CIN3 in the ATHENA (Addressing The Need for Advanced HPV diagnostics) trial [53].

The detection of multiple HR-HPV infections has become a key issue in the development of cervical lesions and the epidemiological status of the population, which is why more efficient genotyping strategies are needed. Different studies have reported that multiple HR-HPV acted synergistically in cervical carcinogenesis [54, 55], and cancers with multiple HPV infections could be more resistant to therapy than those with a single infection [56]. On the other hand, Schmitt et al found that the occurrence of multiple HPV infections did not affect the risk of a lesion being high- or low-grade [57], and Wentzensen et al reported no association between disease status and the number of genotypes detected in a woman [31].Previous works showing an increased risk of CIN with multiple HPV infections had few CIN2+ cases, and restricted to younger women [58], a subgroup known to harbour a larger number of HPV infections [59].

In our study, a significant larger percentage of coinfections was observed in ≤CIN1 (85%) compared to CIN3+ (41%). Such a high amount of multiple infections has important implications to estimate the fraction of low and high-grade cervical abnormalities that are causally attributable to anyone, or a group, of HPV types. Moreover, as clearly shown by the zoom inside the heat map (Fig 4), when CIN3+ histology is compared to ≤CIN1 group in multiple infections cases, there is a strong dominance of HR- genotypes (higher relative proportions in the viral mix). Conversely, in the ≤CIN1 group a marked diversity of genotypes was observed, with none prevailing. Deep amplicon sequencing generated abundant mapped reads and deciphered both the genotype composition within each sample and the proportion of each one in the viral mixture. Our results agree with those of Shen-Gunther et al, who showed that the viral community differed between LSIL and HSIL with a loss of genotypic diversity and domination by carcinogenic HPVs, in particular, HPV-16 in HSIL [25]. HR- HPVs, in particular HPV- 16 and -18, have been shown to be indicators and predictors of CIN3 development [53, 60], being carcinogenic HPV dominance (≥50%) a probable indicator of underlying high-grade disease. These findings are also in line with Depuydt et al, who described in women with multiple HPV types that developed CIN3+, that there was always one of the detected HPV-types present representing a clonal population; the infected basal cell divides with a fixed number of HPV DNA copies per clonal cell, corresponding to an exponential linear growth over time [28]. These metagenomic characteristics are consistent with the ecological principles of competitive exclusion and carcinogenesis hallmarked by clonal expansion and evolution of transformed cells [28, 61, 62]. Distinctive features of altered diversity and dominance among HPV communities within the histological spectrum could be used as a biomarker of disease severity.

The comparison of HPV genotypes identified by PCR-RBH and NGS showed good concordance both when genotypes were analysed individually and when compared as a whole group (genotypes detectable by all 37 RBH probes). However, NGS was able to identify eighty-nine new positive results for HPV genotypes not detected by PCR-RBH, evidencing NGS’ greater sensitivity. The lesser detection capability of PCR-RBH may be partially due to the presence of a viral variant not covered by the probes, or to cross reactivity with another type present in the same sample [63].

In agreement with previous reports, we noticed that NGS was more sensitive than PCR-RBH, particularly in detecting HPV16, HPV31, HPV35, HPV39 and HPV68 [42, 64, 65]. Among them, HPV16 and HPV31, which are phylogenetically related (gender alpha papillomavirus, species 9), have a high epidemiological and pathogenic relevance, given their leading role in the development of cervical precancer and cancer [60]. NGS gives as advantage a massive and parallel sequencing of single molecules from small amounts of DNA, whose benefit increases when the reaction contains multiple HPV types and primers [66]. The high rate of multiple infections may partly explain the discrepant results observed in the present work.

Additionally, NGS allowed to identify five genotypes (HPVs 30, 62, 74, 87 and 90), beyond the 37 probes included in the RBH assay. Although these genotypes have no proven association with cervical carcinogenesis, it is important to detect them to expand their characterization and add information related to HPV epidemiology and virological surveillance.

On the other hand, RBH yielded eight positive results for HPVs 58 and 59 (HR-HPVs) and HPVs 73 and 82 (probable HR-HPV), which were not obtained by NGS. They may represent occasional false-positive results generated by a cross-reaction that has been described for hybridization-based assays [26].

Our study has several strengths, including the implementation of NGS, an advanced technology for HPV genotyping, to expand knowledge on the distribution and relative proportions of each HPV genotype within a sample, in a series of histologically diagnosed cervical samples, in the context of a screening and triage trial in Latin American women. The study has limitations as well; it was a cross-sectional study in a sub-cohort selected by convenience sampling and cannot be claimed to represent the wider screening population.

In conclusion, NGS is a very sensitive and high-throughput tool that may be useful for HPV epidemiological surveillance and for clinical purposes. It may expand the range of genotypes identified in a sample aliquot in a single run. Furthermore, its high detection capability of an expanded range of HPV genotypes and its ability to determine the proportion of each genotype in coinfections allows for an accurate measure rather than a nominal “positive vs negative” measure for a given HPV genotype. In severe lesions, the drop of genotypic diversity and/or the increase of the relative proportion of some of the HR-HPVs in multiple infections could be applied to the follow-up of patients as a biomarker of potential risk for malignant progression.

## Supporting information

S1 Table HPV genotyping by NGS: Reads data. (XLSX)
